# Supplementary figures and images for: Key determinants of the dual clamp/activator function of Complexin
Source: eLife. 2024 Nov 25;12:RP92438. doi: 10.7554/eLife.92438 (PMC11589869; doi:10.7554/eLife.92438)

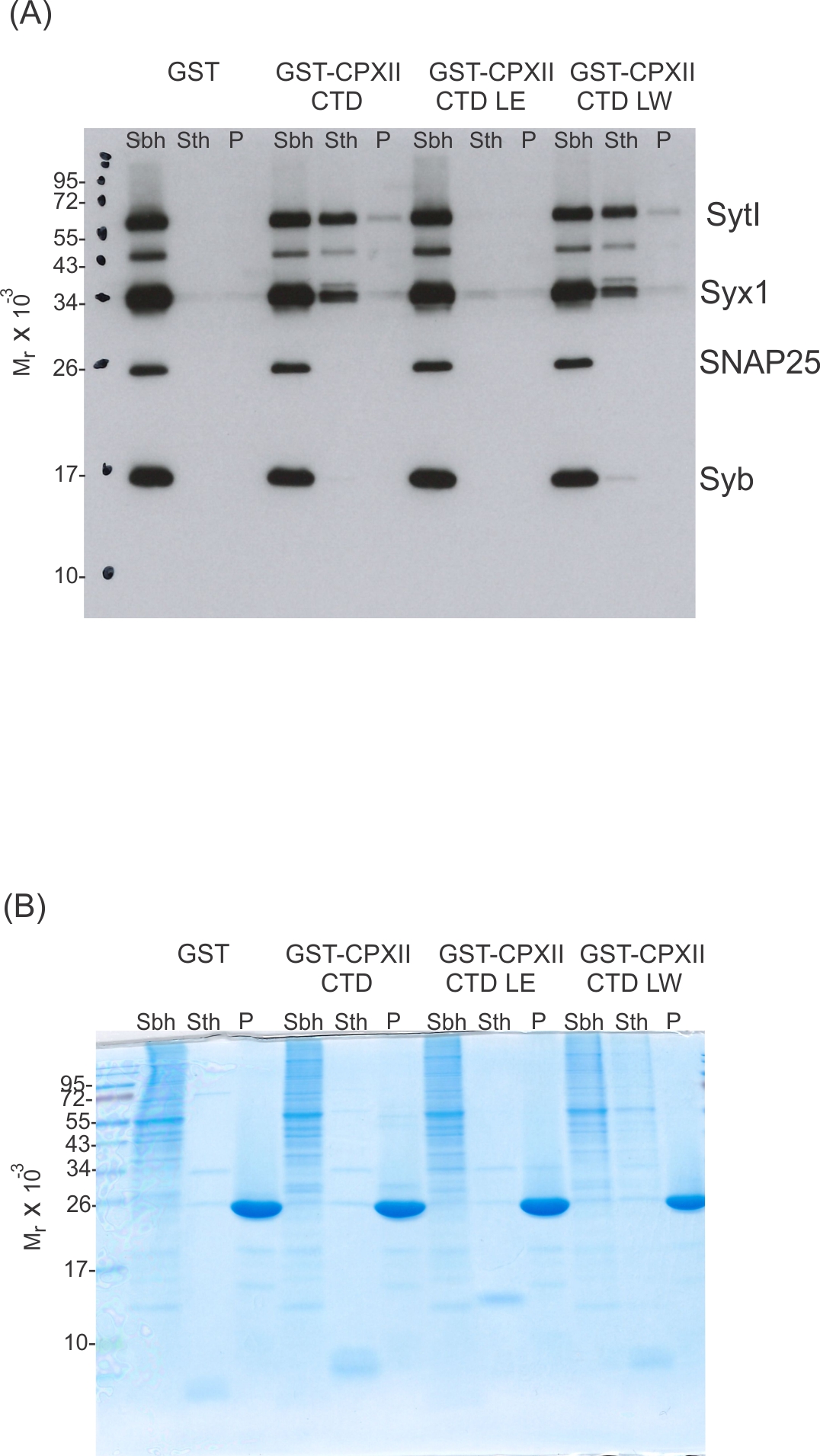

Supplement: Figure 3—source data 1. [file elife-92438-fig3-data1.jpg]

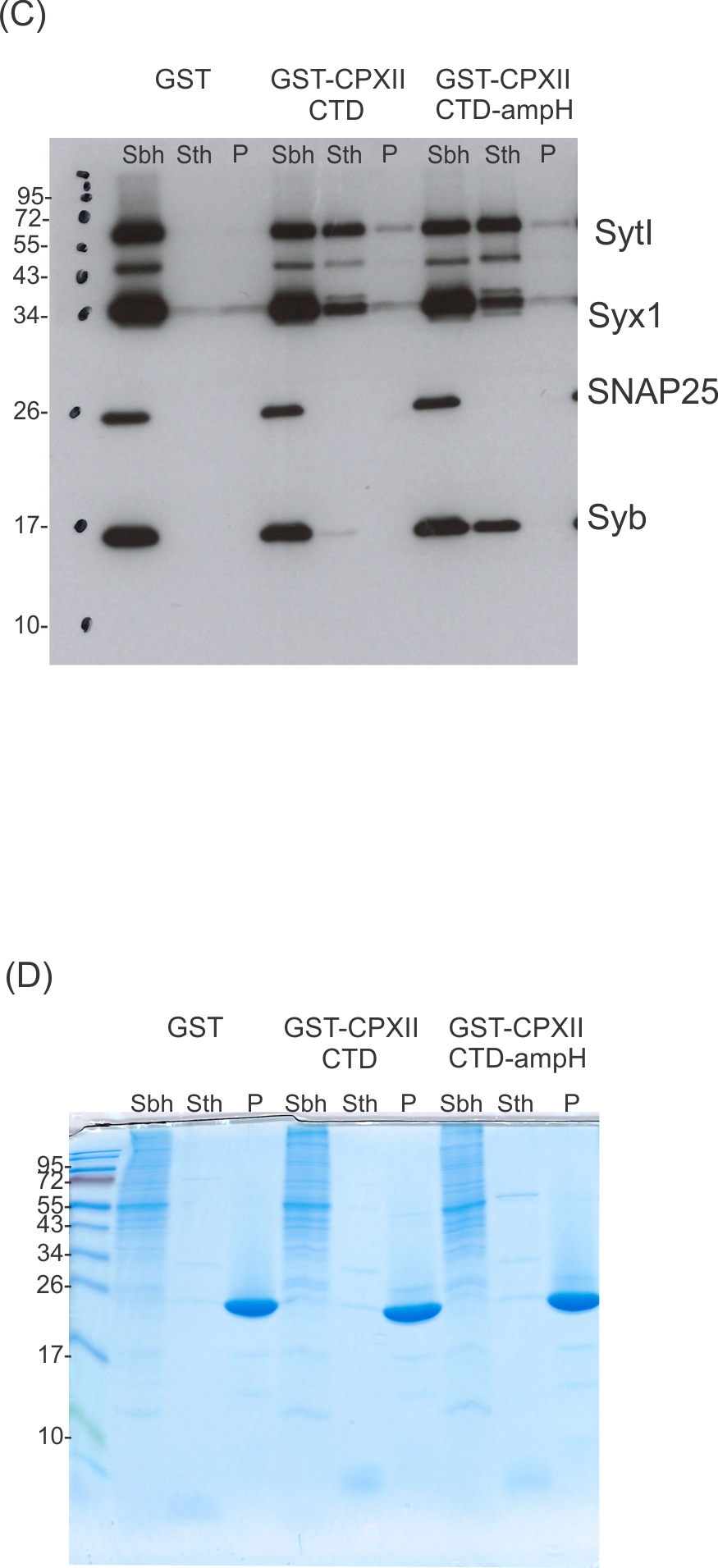

Supplement: Figure 3—source data 2. [file elife-92438-fig3-data2.jpg]
